# The perceived organizational impact of the gender gap across a Canadian department of medicine and proposed strategies to combat it: a qualitative study

**DOI:** 10.1186/s12916-018-1032-8

**Published:** 2018-04-10

**Authors:** Reena Pattani, Christine Marquez, Camellia Dinyarian, Malika Sharma, Julie Bain, Julia E. Moore, Sharon E. Straus

**Affiliations:** 1 Division of General Internal Medicine, St. Michael’s Hospital, 30 Bond Street, Toronto, ON M6H2X5 Canada; 2 Department of Medicine, University of Toronto, Toronto, ON M5G2C4 Canada; 3 Knowledge Translation Program, Li Ka Shing Knowledge Institute, St. Michael’s Hospital, 209 Victoria St, Toronto, ON M5B 1T8 Canada; 4 Maple Leaf Medical Clinic, Toronto, ON M5G1K2 Canada; 5 Casey House, Toronto, ON M4Y1P2 Canada; 6 Division of Geriatrics, St. Michael’s Hospital, 30 Bond Street, Toronto, ON M6H2X5 Canada; 7 Institute of Health Policy, Management, & Evaluation, University of Toronto, Toronto, ON M5T3M6 Canada

**Keywords:** Gender gap, Equity, Academic medicine, Healthcare human resources, Organizational effectiveness, Workplace culture

## Abstract

**Background:**

Despite the gender parity existing in medical schools for over three decades, women remain underrepresented in academic medical centers, particularly in senior ranks and in leadership roles. This has consequences for patient care, education, research, and workplace culture within healthcare organizations. This study was undertaken to explore the perspectives of faculty members at a single department of medicine on the impact of the existing gender gap on organizational effectiveness and workplace culture, and to identify systems-based strategies to mitigate the gap.

**Methods:**

The study took place at a large university department of medicine in Toronto, Canada, with six affiliated hospitals. In this qualitative study, semi-structured individual interviews were conducted between May and September 2016 with full-time faculty members who held clinical and university-based appointments. Transcripts of the interviews were analyzed using thematic analysis. Three authors independently reviewed the transcripts to determine a preliminary list of codes and establish a coding framework. A modified audit consensus coding approach was applied; a single analyst reviewed all the transcripts and a second analyst audited 20% of the transcripts in each round of coding. Following each round, inter-rater reliability was determined, discrepancies were resolved through discussion, and modifications were made as needed to the coding framework. The analysis revealed faculty members’ perceptions of the gender gap, potential contributing factors, organizational impacts, and possible solutions to bridge the gap.

**Results:**

Of the 43 full-time faculty members who participated in the survey (29 of whom self-identified as female), most participants were aware of the existing gender gap within academic medicine. Participants described social exclusion, reinforced stereotypes, and unprofessional behaviors as consequences of the gap on organizational effectiveness and culture. They suggested improvements in (1) the processes for recruitment, hiring, and promotion; (2) inclusiveness of the work environment; (3) structures for mentorship; and (4) ongoing monitoring of the gap.

**Conclusion:**

The existing gender gap in academic medicine may have negative consequences for organizational effectiveness and workplace culture but many systems-based strategies to mitigate the gap exist. Although these solutions warrant rigorous evaluation, they are feasible to institute within most healthcare organizations immediately.

## Background

Investigating the barriers faced by women in academic medicine is a century-old endeavor [1], but gender disparity persists. Women remain underrepresented in academic medical centers [2], particularly in senior and leadership positions, despite enrollment in medical schools hovering at 50% since the 1980s [3]. Since 2008, the Association of American Medical Colleges (AAMC) has been administering the Women in Medicine and Science (WIMS) survey to track the progress of women in medicine through the academic life cycle [3]. The findings are noteworthy: the number of female full professors in United States academic medical centers increased from 14% to 22% between 2009 and 2014, a gain that nonetheless falls short based on the gender balance of medical school graduates over the past four decades. Furthermore, the percentage of newly tenured women over the same period stagnated at 30% [3]. Women comprised only 15% of department chairs and 16% of deans [3]. A recent cross-sectional analysis showed that only seven out of 50 National Institutes of Health (NIH)-funded institutions had more than 20% female department leaders [4]. There are fewer women than men serving as lead authors of publications in top medical journals [5], presenting at medical grand rounds [6], and participating in clinical practice guidelines committees [7]. The dearth of women in academic medicine and science has been documented across the globe [8–12].

Evidence suggests that women and men share similar career aspirations in medicine [13], and that gender does not predict attrition from academic medicine [14]. Findings suggest that the work environment may be responsible for poor rates of retention of women in academic medicine, a phenomenon colloquially referred to as the “leaky pipeline” [15]. The lack of gender parity in medicine and academia has been attributed to several factors including inequity in pay for equal work [16], differences in securing funds for research [17], lower teaching evaluations due to gender bias [18], lower recruitment, hiring, and mentoring of women due to unconscious bias [19], inadequate recognition for work performed [20, 21], less access to mentorship and sponsorship [22, 23], and external factors like care responsibilities at home that place greater demands on women [24]. These factors may result in women being pushed out rather than opting out of successful careers in academic medicine [25].

The impact of the existing gender gap on the organizational effectiveness and workplace culture of academic medical centers has yet to be explored. Furthermore, there is scant evidence that supports interventions to counter the gender gap in academic medicine [26–28]. Academic physicians may have suggestions regarding potential strategies to bridge this gap based on their experiences.

## Methods

### Aim

The purpose of this paper is to explore faculty members’ perspectives regarding the organizational impact of the gender gap and to identify potential strategies that could be implemented to promote gender equity in a large university-based department of medicine.

### Setting

The Department of Medicine (DOM) at the University of Toronto is composed of 800 full-time faculty members, 1000 postgraduate trainees, and 19 sub-specialty divisions located across six fully affiliated hospitals. Unpublished data from a faculty survey conducted within the DOM at the University of Toronto in 2015 suggested that the representation of women at this institution is comparable to the representation noted in the WIMS survey [29]: 36% of full-time faculty within the university DOM are women and 25% of full professors are women. These findings persist even though men and women enter the junior ranks at our university in approximately equal numbers.

### Study design

We used the consolidated criteria for reporting qualitative research (COREQ) to design and report this study [30]. We conducted a qualitative study using semi-structured individual interviews with faculty members from the DOM at the University of Toronto.

### Sampling and recruitment

Our local data from the 2015 faculty survey suggested that there were gender differences according to faculty academic position description, academic rank, and hospital. Thus, we used a purposive sampling strategy to identify DOM members across various academic positions (i.e., clinician-teacher, clinician-educator, clinician-investigator, clinician-scientist, clinician-administrator, and clinician-quality and innovation specialist). These academic position descriptions vary according to the time spent in clinical service, the nature of the scholarly work (Table 1), and their promotion requirements [31]. We also identified participants across academic ranks (i.e., lecturer, assistant professor, associate professor, and full professor) and hospitals. We aimed to recruit participants until we achieved a saturation of themes [32]; we anticipated the need to recruit one or two individuals from each of the six fully affiliated hospitals by academic position description to reach this goal [33].

**Table 1:** Academic position descriptions within the Department of Medicine, University of Toronto

Academic position	Target percentage of professional time in clinical care	Academic focus
Clinician-teacher	60–75%	Teaching and clinical care
Clinician-educator	30–50%	Education research, leadership, and administration
Clinician-investigator	30–50%	Research and related CPA^a^
Clinician- scientist	10–25%	Research (all types)
Clinician-administrator	0–25%	Senior leadership role at faculty, university, or hospital level
Clinician-quality and innovation specialist	60–75%	Quality improvement, patient safety, and healthcare innovation (includes teaching, research, and CPA)

^a^CPA refers to creative professional activities, which are activities that demonstrate impact outside of the traditional clinical, research, or education domains [59]

We obtained a list of full-time faculty members within the DOM and a member of the research team sent email invitations describing the study to potential participants. To maximize the response rate, an email reminder was sent. We also employed a snowball sampling strategy, wherein participating faculty members were asked to support recruitment by identifying other DOM members who might be willing to be interviewed.

### Data collection

We conducted a series of one-on-one semi-structured interviews between May and September 2016. We developed questions based on our 2015 survey results and feedback from the research team. The questions included inquiries around perceptions of the gender gap, factors contributing to the gender gap, the impact of the gap on organizational effectiveness and workplace culture, and possible strategies to mitigate it. The interview guide is in given in the Appendix. The interviews were conducted by telephone, and lasted 45 to 60 min. They were recorded, transcribed verbatim, and de-identified. The units of analysis included the transcripts as well as field notes taken by the research assistant during the interviews. We collected participant demographic information including gender, hospital, academic position, academic rank, division, and stage of career (denoted by years of service). We asked participants for permission to use de-identified quote(s) prior to inclusion in the report and/or publication. We provided participants with a draft of the report for review.

### Data analysis

We analyzed transcripts using a thematic analysis approach [34] with NVivo 11 software [35]. Three members of the research team (CM, CD, and RP) independently reviewed a portion of the transcripts with the accompanying field notes to develop a preliminary list of codes based on impressions of recurring themes. The analysts then convened to compare their interpretations of the data and formed an initial coding framework, reviewing it for clarity and comprehensiveness. We applied a modified audit consensus coding approach [36]: two analysts (CM and CD) independently piloted the coding framework by applying it to two transcripts and used NVivo 11 software to determine inter-rater reliability, which was calculated using the kappa coefficient. Discrepancies were resolved by discussion, and modifications were made to the coding framework as needed, until a kappa coefficient ≥ 0.6 was obtained. The remaining transcripts were divided and coded in three sequential rounds, where each round consisted of 1/3 of the remaining transcripts (less the two in the pilot). For each round of coding, the primary analyst (CM) coded all the transcripts and a secondary analyst (CD) independently coded 20% of the transcripts as an audit. After each round, we again assessed inter-rater reliability, resolved discrepancies by discussion until the kappa coefficient was ≥ 0.6, and made changes to the framework as necessary, prior to advancement to the next round. We used written memos and annotations to record the analytic process and to form a basis for the propositions that we developed.

### Ethics and consent

We obtained ethics approval from the Research Ethics Boards of the University of Toronto (REB 32726) and St. Michael’s Hospital (REB 15–273) in Toronto, Canada. Informed consent was obtained verbally from all participants and recorded prior to the interviews.

## Results

We interviewed 43 full-time faculty members. Of these, 29 (67%) participants self-identified as female and 14 (33%) as male. There were between 1 and 14 participants from each of the six fully affiliated teaching hospitals. We had representation from the following divisions: Cardiology, Critical Care, Emergency Medicine, Endocrinology, Gastroenterology, General Internal Medicine, Geriatric Medicine, Infectious Diseases, Medical Oncology, Nephrology, Respirology, and Rheumatology. We were unable to recruit participants from Clinical Immunology and Allergy, Clinical Pharmacology, Dermatology, Hematology, Neurology, Occupational Medicine, and Physical Medicine and Rehabilitation. Further demographic information is listed in Table 2. To preserve participant anonymity, we are unable to present full demographic details.

**Table 2:** Demographic variables

Demographic variable	Number of participants (percentage)
Gender	
Female	29 (67%)
Academic position	
Clinician-teacher	10 (23%)
Clinician-educator	5 (12%)
Clinician-investigator	8 (19%)
Clinician-scientist	8 (19%)
Clinician-administrator	5 (12%)
Clinician-quality improvement specialist	6 (14%)
Preferred not to say	1 (2%)
Academic rank	
Lecturer	3 (7%)
Assistant professor	19 (44%)
Associate professor	3 (7%)
Full professor	17 (40%)
Preferred not to say	1 (2%)
Stage of career	
Early career (< 5 years)	11 (26%)
Mid-career (5–15 years)	16 (37%)
Late career (> 15 years)	16 (37%)

Most participants were not surprised by the presence of a gender gap across the DOM and their professional experiences corroborated the empirical data. We identified participants’ perceptions of the impact of the existing gender gap on organizational effectiveness and workplace culture, as well as suggestions to combat the existing gap.

### Organizational impact of the gender gap

The participants perceived that there were several impacts on organizational effectiveness and workplace culture associated with the gender gap at our DOM including social exclusion, reinforced stereotypes, and unprofessional behavior.

#### Social exclusion

Participants correlated a lack of gender diversity in the work environment with segregation in both formal and informal professional activities. Women felt excluded from certain informal networking activities, including recreational sporting activities, meetings at bars, and weekend trips. Although male participants acknowledged a separation between men and women, they did not feel personally or professionally affected by the segregation. In contrast, women attributed a “*lingering old boys’ club mentality*” with reduced opportunities for career advancement. As one female participant noted, “*You were not participating that much in that informal networking after hours. And, so, in that way, it could have impacted you, because it didn’t show all your skills that could, you know, have put you more in the leadership position at an earlier age.*”

Female participants also felt that in a male-dominated work environment, there was often a lack of consideration for the disproportionate responsibility carried by women at home, with adverse consequences for their ability to thrive in the workplace. For instance, women felt that the scheduling of meeting times outside of regular business hours made it difficult for them to actively participate in workplace governance and to take on leadership positions: “*I think the real difference is that most of my male colleagues can stay late at night, because their wives are home to put the kids to bed, but for women — like, where does that happen?*” Furthermore, participants felt that in a male-dominated work environment, the ambiguity and lack of support around parental leave was maintained as the status quo.

#### Reinforced stereotypes

Participants felt that there were differences in gender parity across specialties, with more women represented in certain specialties like rheumatology, and less women in others like cardiology. This was felt to be problematic because of the possibility that trainees and future recruits might view certain disciplines as being unfavorable for women, as noted by one female participant:

It’s potentially sending the message that it’s not a conducive career for young women, even if that is not the reason, you know, if it has nothing to do with why people do or don’t choose to work in clinically intensive areas. … Where are all the women with young families? Maybe that is not the place for me, or maybe it’s just not possible*.*

Interestingly, there was a perception that there were more women compared to men working in certain academic positions like clinician-teacher. Participants of both genders speculated that more women were drawn to this academic position because it does not require further graduate training. This was a misperception given that all academic positions within our DOM require additional postgraduate education.

#### Unprofessional behavior

Many participants reported observing or experiencing unprofessional behavior, with some participants correlating this with the gender gap. Unprofessionalism was manifested through inappropriate comments (e.g., commenting on an individual’s appearance or size), the use of gender-based pejorative language, or interruptions and over-talking during meetings. These behaviors led to a perceived undervaluing of female faculty, with negative consequences for culture, engagement, and workforce retention. As one female participant noted, “*I think that there are instances of disrespect shown to women that have made them withdraw or move somewhere else or just refuse to participate because of their anger and fear*.” Some of the participants also felt that complaints about unprofessionalism were at risk of being dismissed: “*It’s still a man’s world. It’s still ok to complain about women in a way that it’s not ok to complain about men or that no one would ever complain about men*,” observed a male participant. Not all participants correlated increased rates or severity of unprofessional behavior with the gender gap. Some participants wondered whether these behaviors were attributable to a generational gap or cultural differences.

### Proposed interventions to combat the gender gap

Participants offered several suggestions for reducing the gender gap including improvements in recruitment, hiring, and promotion practices; cultivation of a supportive and inclusive work environment; improvement in mentorship opportunities; and ongoing monitoring and examination of the gender gap.

#### Recruitment, hiring, and promotion practices

Although all participants agreed that substantial gains had been made toward improving equity in recruitment and hiring within our DOM, several participants advocated for additional changes to overcome the impact of residual gender bias. For instance, they felt that the job recruitment process was still highly contingent on informal networking, which was perceived to disadvantage women. They suggested increasing transparency through formalized search processes for job openings, with clear and widely distributed job listings. One male participant remarked:

I think if we made that recruitment prospective, strategic, and formal, it would at least call out some of those biases. So, if we said as a division in 2 years or 3 years we would like to recruit X type of clinician … that would provide an opportunity for everyone to throw potential candidates into the pool*.*

Some participants referenced interventions to promote equity being used in other sectors, like hosting networking events specifically for women. These events might include seminars for skills development on topics like negotiation or leadership. With regard to promotions, participants felt that there needed to be a re-examination of the approaches (e.g., timelines) and criteria (e.g., number of grants) for academic promotion, given that existing processes may disadvantage women, particularly those taking parental leave. Several participants emphasized that the goal of these interventions should be to recruit, hire, and promote women at the same rate as men based on fairness, not based on gender.

#### Improvements to the work environment

Although most participants described a supportive work environment, they provided several suggestions of potential strategies to improve the workplace culture. For instance, they suggested that training programs, including unconscious bias training and gender sensitivity training, be made mandatory for individuals in leadership positions—if not all faculty members. There were suggestions to enact a policy stating that meetings should be held during regular business hours to increase the ability of female faculty to participate in governance matters. They also suggested creating systems to support faculty members during career transitions, particularly during return-to-work transitions following parental leave. Participants highlighted the potential use of alternative work arrangements like job-sharing, part-time work, or flexible work hours. Lastly, the establishment of peer-to-peer counseling programs was recommended by participants to address concerns related to equity and diversity in the DOM.

#### Mentorship

Participants perceived a mentorship vacuum within the DOM, which might have a disproportionate impact on women given their historical exclusion from informal networking opportunities. “*It’s almost thought it’s easier to see potential in a man if you’re a senior mentor person [male], than to see the potential in a woman*,” said one female participant. As such, participants suggested that the DOM formalize their mentorship programs, recruiting more senior-level female and male faculty members to serve as mentors and making expectations about roles and responsibilities clear to both mentors and mentees. There were also suggestions to create a formal reward system for mentorship, identifying and celebrating effective mentors. Participants emphasized that mentorship programs should begin early (i.e., with trainees). Participants of both genders had been successfully mentored by both male and female faculty, but some participants wondered if matching mentors and mentees by gender might narrow the gap. As one male participant observed: “*I think the lack of adequate number of female mentors has maybe suggested that a number of female candidates have been mentored by males. Not that that’s wrong, but it may not have met all of their needs.*”

#### Ongoing monitoring and examination of the gender gap

Most participants agreed that efforts should be made to track the gender gap within the DOM over time. They suggested that data be collected prospectively to continue to follow gender trends in recruitment, hiring, and promotions practices. Several participants viewed qualitative data collection as a necessary component of monitoring the gender gap to explore deeper issues.

## Discussion

We sought to explore the perceived impact of the gender gap on organizational effectiveness and workplace culture in academic medicine and to search for possible interventions to counter the existing gap. Participants perceived that social exclusion, reinforced stereotypes, and unprofessional behavior were consequences of a lack of gender diversity across the DOM. These consequences have direct and cascading effects on workplace culture, patient care, and the effectiveness of collaboration in research and education. Put simply, they waste skills and talent, and threaten the effectiveness of healthcare organizations. To address the gender gap, they suggested improvements in processes for recruitment, hiring, and promotion; changes to the work environment; formalization of mentorship opportunities; and ongoing monitoring of the gap.

Many hospitals, universities, and professional bodies—including the AAMC and funding agencies such as the NIH and the Canadian Institutes of Health Research—are increasingly making diversity and inclusion efforts a priority [37–39]. While there are many dimensions of diversity, gender disparities in academic medicine have been found to be a locally [29] and globally [40] pressing issue.

The relationship between gender disparity in academic medicine and workplace culture has been previously explored, although the evidence base is scant. One study [13], which surveyed faculty members across 26 U.S. medical schools, found that women compared to men had a lower sense of belonging, perceived fewer career opportunities, and had less congruence of personal and institutional values despite experiencing equal levels of institutional engagement and aspirations toward leadership. Another survey study found that improved gender equality in academic medicine mitigated burnout among female faculty, although this study was conducted at a single center in Japan [41]. Another group of investigators developed and validated a tool to measure a culture’s conduciveness to women’s academic success (CCWAS) along four dimensions: equal access, work–life balance, freedom from gender biases, and supportive leadership [42], which could be used in future studies exploring this relationship.

There is some evidence to suggest that the existing gender disparity might have implications for patient care, with one large observational study showing reduced hospital mortality and readmission for patients cared for by a female internist compared to a male internist [43]. This suggests that not only does the existing gender gap make little sense for patient care, but also that there is much to be gained from understanding the skills that conferred the mortality difference and designing curricula to cultivate these skills in all medical trainees, regardless of gender. A lack of diversity in academic medicine may also have implications for public health. For instance, racial and ethnic minority groups may be more likely to take up issues that are pertinent to their communities [44, 45]. Given that women are less likely to receive evidence-based tests and interventions [46–49], and they remain underrepresented in clinical trials [50], gender may play a similarly modulating effect on the practice of clinicians and researchers. A group of international scholars have suggested that gender be used explicitly as a variable in judging research impact assessment to serve as a corrective against decades of bias toward male researchers and, consequently, the predominantly male beneficiaries of science and policy [51]. Creative solutions like this require rigorous evaluation.

In academic medicine, the absence of gender parity has a compounding effect because it reduces opportunities for female mentorship, sponsorship [22, 23], and role modeling, with downstream consequences for trainees. Our data suggested there is a perception that there are more women than men in clinician-teacher academic positions. In reality, although this academic position does indeed have the greatest representation of women, women remain outnumbered by men as clinician-teachers across our DOM [29]. It was unclear if the misperception we identified reflects a more widespread perception of teaching as a feminized occupation [52]. Regardless, the false perception that there are more women working as clinician-teachers within our DOM might dampen calls for action to promote equity.

Suggestions from our participants on strategies to narrow the gender gap echo the insights of leaders in academic medicine [38]. The interventions to promote gender equity that have the widest evidence base include gender bias and sensitivity training [53, 54]. Although this evidence includes a cluster randomized controlled trial, these studies rely largely on self-reported outcomes rather than objective measures. Nonetheless, the use of education interventions, particularly for leaders in academic medicine, was also advocated by our participants. Bias and sensitivity training continue to be perceived as a mainstay of diversity efforts across a variety of organizations despite a dearth of evidence that they result in durable change [55]. Indeed, there are concerns that these programs may actually activate bias as a result of the negative framing inherent to existing programs, although this is based on limited evidence, with a paucity of randomized trials, from sectors outside of healthcare [55, 56]. Critically, our participants also recommended concomitant structural changes to the workplace that may improve women’s access to informal networking, mentorship, opportunities for advancement, and the ability to participate in governance matters.

Our study adds to the existing literature on the impact of the gender gap on workplace culture by using qualitative methods to explore frontline perspectives. To our knowledge, ours is the first study hypothesizing a link between the gender gap and a higher prevalence of unprofessional behaviors. Our observation that the gender gap in academic medical centers may contribute to segregation and the perpetuation of stereotypes may hint at some of the pathways that lead to the cultural reproduction of male dominance in academic medicine, despite the gender parity among medical school graduates over several decades. A strength of our study is that it was conducted within a large DOM [29] that can be considered representative of academic medical centers in other contexts [3]. Another strength is that the systems-based strategies advanced by our participants are feasible to institute immediately, and at low cost, in most healthcare organizations.

There were several limitations in this study. Firstly, participation may have been limited to those individuals who perceived the existing gender gap to be a problem. We tried to minimize this by reaching out to all members of our department with an email invitation to participate in addition to using a snowball sampling strategy. Ultimately, we had a relatively large sample for a qualitative study and included individuals with diverse opinions as reflected in the results. We were unable to recruit participants from seven of the subspecialties of medicine, likely due to their small size; however, the represented divisions span both the procedural and cognitive subspecialties of medicine and likely capture the diversity of work microenvironments and cultures. Finally, many of the assertions made through our data collection used a heteronormative frame of reference. Indeed, we did not take an intersectional approach nor look at other demographic variables like sexual orientation, race, or ethnicity [57]. A key challenge in understanding the gender gap from an intersectional perspective is the current underrepresentation of women, racialized people, and LGBTQ individuals in academic medicine. This underrepresentation can make it logistically challenging to identify participants and potentially risky for participants to speak out for fear of being identified or penalized. There is an emerging evidence base suggesting that the interplay of these factors creates even greater barriers for career advancement in academic medicine [58], and this certainly warrants further exploration.

## Conclusions

The existing gender gap in academic medical centers may have negative consequences for organizational effectiveness in patient care, research, and medical education, as well as workplace culture. Strategies like standardizing processes for recruitment, hiring, and promotion; intervening on workplace culture directly with education and training; and strengthening mentorship opportunities, warrant dedicated and rigorous evaluation. Ongoing monitoring of the gap will permit us to document progress over time.

## Appendix

**Table 3:** Gender gap interview guide

Interview questions	Interviewer’s notes
Background Information:	
I have some information that I’d like to share before I asked you the first question. So for the past 10 years, the proportion of men has consistently been higher than the proportion of women in such positions as scientists, teachers, educators, and investigators within the University Of Toronto, Department Of Medicine (UofT DOM). For example, statistics gathered from the U of T DOM in 2015 reveal that 41.8% of women hold a full or associate professor position while 55 % of men hold a full or associate professor position.	
1. What is your impression with respect to the information that I’ve read regarding the disproportion or gender gap amongst DOM members? (i.e., were you surprised to hear about this information/not surprise to hear about this information)	
If surprised/or in disagreement with statistics –	
So you mentioned that you are (surprise/don’t agree) with the statistics, can you explain why (i.e., do you see a more equal distribution of women and men in your workplace)? Are there specific examples of actions that you have seen to reduce the gap between women and men in the DOM?	
So having heard about the gender gap, can you hypothesize as to why it might exist in the DOM? OR skip to question #2.	
Probe:	
a. What are some of the roles that women have in your workplace (i.e., researcher or scientist)?	
2. In your opinion, what are some of the reasons this gender gap exists in the DOM? OR what would you speculate are some of the reasons this gender gap exists in the DOM?	
Are there any other factors that you feel influence this gender gap?	
Probes:	
a. Are you aware of the recruitment approaches in the DOM? If so, what is your perception of how members of the DOM are hired at UofT?	
b. May I ask…how were you hired?	
3. Do you think the gender gap has influenced the work environment at the DOM? (for example the norms or culture at the DOM)	
*If interviewee is having difficulties answering this question then consider re-phrasing and provide example:*	
Can you describe the works place environment (i.e., what are the norms or culture at the DOM amongst women and men, is there any differences between gender?) For example do you feel that women receive the same support as men in the DOM	
Do you feel supported in your work environment?	
I would now like to move away from the DOM and ask you some questions about your hospital setting.	
4. In your opinion, do you feel that there is a gender gap within your hospital division? (i.e., is there a disproportion of women versus men in your hospital?)	
• If yes, probes:	
a. How so and can you provide specific examples?	
b. In your opinion, what are some of the reasons this gender gap exists within your hospital division?	
i. Are you aware of the recruitment approaches at within your hospital division? If so, what is your perception of how staff are hired within your hospital division?	
ii. May I ask…how were you hired?	
c. Do you think the gender gap has influenced the work environment within your hospital division?	
d. Do you feel that there is a gender gap at other hospital divisions? If yes, how so and can you provide specific examples?	
• If no (i.e., they do not feel that there is a gender gap or they are unaware of a gender gap within their hospital division) probes:	
a. What are some of the actions that your hospital division has taken that you feel eliminates the perception that there is a gender gap amongst staff?	
b. Was there ever a situation in which you felt that perhaps gender gap was a factor? If yes, how so and can you provide examples?	
c. Do you feel that there is a gender gap at other hospital divisions? If yes, how so and can you provide examples?	
I would now like you to reflect on your impressions of both the DOM and your hospital setting for the next couple of questions I have for you…	
5. What is your experience with career mentorship at the DOM and within your hospital division?	
Probes:	
a. Did you have a career mentor?	
b. Have you had the opportunity to be a career mentor to others?	
c. Are you aware if there is a current mentorship model at the DOM or within your hospital division?	
d. In your opinion, has the presence of mentors/mentee relationship influenced the proportion of women versus men in your workplace environment?	
6. Have you been personally impacted by the gender gap? (i.e., have you benefited or placed at a disadvantage)	
• If yes, probe:	
a. How so and can you provide examples?	
b. Do you know of anyone who has been personally impacted? Can you describe the situation/outcome?	
• If no, probe:	
a. Can you describe why you think you have not been impacted? Can you provide examples?	
b. Do you know of anyone who has been personally impacted? Can you describe the situation/outcome?	
7. In your opinion, hearing about these statistics and that there is a gender gap present at DOM, how should the gender gap be addressed (if at all)?	
8. Do you believe that the gender gap should be monitored over time?	
a. If yes, how so? (probe for suggestions)	
9. Is there any other feedback that you wish to include about gender gap that we have not discussed?	
